# Effects of Positive End-Expiratory Pressure on Pulmonary Oxygenation and Biventricular Function during One-Lung Ventilation: A Randomized Crossover Study

**DOI:** 10.3390/jcm8050740

**Published:** 2019-05-23

**Authors:** Namo Kim, Su Hyun Lee, Kwan Woong Choi, Haeyeon Lee, Young Jun Oh

**Affiliations:** 1 Department of Anesthesiology and Pain Medicine, Anesthesia and Pain Research Institute, Yonsei University College of Medicine, 50-1 Yonsei-ro, Seodaemun-gu, Seoul 03722, Korea; namo@yuhs.ac; 2 Department of Anesthesiology and Pain Medicine, Yonsei Sarang Hospital, 10, Hyoryeong-ro, Seocho-gu, Seoul 06698, Korea; goodieoh@gmail.com; 3 Department of Anesthesiology and Pain Medicine, National Health Insurance Service Ilsan Hospital, 100, Ilsan-ro, Ilsandong-gu, Goyang-si, Gyeonggi-do 10444, Korea; sanctum@nhimc.or.kr (K.W.C.); hylee1231@yuhs.ac (H.L.)

**Keywords:** biventricular function, one-lung ventilation, positive end-expiratory pressure, pulmonary oxygenation

## Abstract

Although the application of positive end-expiratory pressure (PEEP) can alter cardiopulmonary physiology during one-lung ventilation (OLV), these changes have not been clearly elucidated. This study assessed the effects of different levels of PEEP on biventricular function, as well as pulmonary oxygenation during OLV. Thirty-six lung cancer patients received one PEEP combination of six sequences, consisting of 0 (PEEP_0), 5 (PEEP_5), and 10 cmH_2_O (PEEP_10), using a crossover design during OLV. The ratio of arterial oxygen partial pressure to inspired oxygen fraction (P/F ratio), systolic and diastolic echocardiographic parameters were measured at 20 min after the first, second, and third PEEP. P/F ratio at PEEP_5 was significantly higher compared to PEEP_0 (*p* = 0.014), whereas the P/F ratio at PEEP_10 did not show significant differences compared to PEEP_0 or PEEP_5. Left ventricular ejection fraction (LV EF) and right ventricular fractional area change (RV FAC) at PEEP_10 (EF, *p* < 0.001; FAC, *p* = 0.001) were significantly lower compared to PEEP_0 or PEEP_5. RV E/E’ (*p* = 0.048) and RV myocardial performance index (*p* < 0.001) at PEEP_10 were significantly higher than those at PEEP_0 or PEEP_5. In conclusion, increasing PEEP to 10 cmH_2_O decreased biventricular function, especially on RV function, with no further improvement on oxygenation compared to PEEP 5 cmH_2_O during OLV.

## 1. Introduction

One-lung ventilation (OLV), which is essential in thoracic surgery, induces ventilation/perfusion ratio (V/Q) mismatch by increasing intra-pulmonary shunts and dead space [1]. Historically, large tidal volumes were applied to prevent unfavorable intraoperative atelectasis and improve gas exchange during OLV [2]. However, several studies have shown that lung injury after thoracic surgery is associated with OLV [3,4]. Therefore, an optimal strategy for OLV is needed not only for maintaining adequate gas exchange, but also for protecting the lung.

Application of positive-end expiratory pressure (PEEP) is an important factor in optimal OLV strategy, and several studies have investigated the amounts of PEEP that are beneficial during OLV. A recent study showed that an “individualized” PEEP level measuring around 10 cmH_2_O improved pulmonary oxygenation during OLV [5]. However, in two-lung ventilation (TLV), aggressive mechanical ventilation using high levels of PEEP exceeding 10 cmH_2_O can restrict venous return and elevate right ventricular (RV) afterload, leading to limited left ventricular (LV) diastolic filling and decreased cardiac output [6].

No existing prospective study has yet evaluated the effect of PEEP on pulmonary oxygenation and biventricular function simultaneously during OLV. The primary purpose of this study was to compare the effects of different levels of PEEP on pulmonary oxygenation and biventricular function in patients undergoing thoracic surgery under OLV.

## 2. Materials and Methods 

### 2.1. Patients

This study was approved by the institutional review board of Yonsei University Health System (IRB # 4-2015-0325) in Seoul, South Korea, and written informed consent was obtained from all patients before participating in the study. The trial was registered prior to patient enrollment at clinicaltrials.gov (NCT02483806). 

For this study, we enrolled lung cancer patients who were scheduled for single lobe lobectomy with video-assisted thoracoscopic surgery (VATS) from September 2015 to April 2016. The inclusion criteria were patients aged from 40 to 80 years and patients with American Society of Anesthesiologists physical status classification score of II–III. The exclusion criteria were patients with any kind of arrhythmia; New York Heart Association functional classification of III–IV; end-stage liver or kidney diseases; esophageal varices; and those who had either severe obstructive or restrictive lung diseases. 

### 2.2. Anesthesia and Procedural Protocols

No premedication was given to any of the patients. Anesthesia was induced using propofol (1.0–2.0 mg/kg), remifentanil (0.5–1.0 μg/kg), and 0.9 mg/kg of rocuronium. All patients were intubated with left-sided double-lumen tubes (35 Fr for women, 37 Fr for men, Broncho-Cath®; Mallinckrodt Medical, Inc., Athlone, Ireland), and correct positioning was confirmed using a fiber-optic bronchoscope. Patients’ arterial blood pressure and central venous pressure levels were monitored using standard monitoring devices. A central vein catheter was inserted at the right internal jugular vein. Multiplane transesophageal echocardiography probes (6TC; GE Vingmed Ultrasound AS, Horten, Norway) were inserted, and lung alveolar recruitment was performed with maximal 30 cmH_2_O of pressure for 30 seconds. During the induction period, 5 mL/kg of Hartmann’s solution was given to each patient to minimize preload reduction by PEEP application. 

Anesthesia was maintained with 1.0–2.0 vol % sevoflurane and 0.05–0.1 μg·kg^−1^·min^−1^ of remifentanil targeted at Bispectral index (BIS VISTA™, Aspect Medical Systems, Norwood, MA, USA) between 40 and 60. Autoflow pressure-controlled ventilation mode (Primus i® ventilator; Dräger™, Lübeck, Germany) was applied to all patients. Ventilation was initiated using a tidal volume of 6 mL/kg of the predicted body weight with 1:2 inspiratory-to-expiratory ratio, an inspired oxygen fraction (FiO_2_) of 0.6, and 5 cmH_2_O of PEEP. All patients had an initial PEEP level of 5 cmH_2_O during TLV. Respiratory rate was adjusted to maintain an end-tidal carbon dioxide (EtCO_2_) at approximately 40 mmHg. When the patient’s oxygen saturation measured by pulse oximetry decreased to less than 90% during OLV, FiO_2_ was incrementally increased to 0.8 and 1.0.

### 2.3. Study Design and Outcome Assessments

PEEP was applied at three different levels of 0 cmH_2_O, 5 cmH_2_O, and 10 cmH_2_O; and the measurements of each PEEP application was designated as PEEP_0, PEEP_5, and PEEP_10, respectively, for statistical analysis. The study had a crossover design with patients randomly assigned to one of six PEEP sequence combinations of 0, 5, and 10 cmH_2_O. The baseline hemodynamic, echocardiographic, and respiratory variables were measured at 10 min after the recruitment maneuver during TLV in a supine position. The patient was then placed in the lateral decubitus position. When hemodynamic parameters were stabilized, OLV was initiated and variables were measured again at 20 min after the first, second, and third PEEP applications. Since vascular clamping can alter perfusion of the non-dependent lung, patients were excluded if the pulmonary vessel was clamped for lobectomy during the experimental period.

Arterial blood gas analyses were performed at every experimental step, and the respiratory variables included FiO_2_, EtCO_2_, ratio of arterial oxygen partial pressure (PaO_2_) to FiO_2_ (P/F ratio), peak airway pressure (Ppeak), dynamic compliance, and dead space ventilation (alveolar dead space/alveolar tidal volume, V_D_/V_T_). Dynamic compliance was calculated as V_T_/(Ppeak−PEEP). V_D_/V_T_ was calculated in accordance to Hardman and Aitkenhead equation [7] using the values of EtCO_2_ and arterial carbon dioxide partial pressure (PaCO_2_) as V_D_/V_T_ = 1.14 × (PaCO_2_−EtCO_2_)/PaCO_2_−0.005.

**Figure 1:**
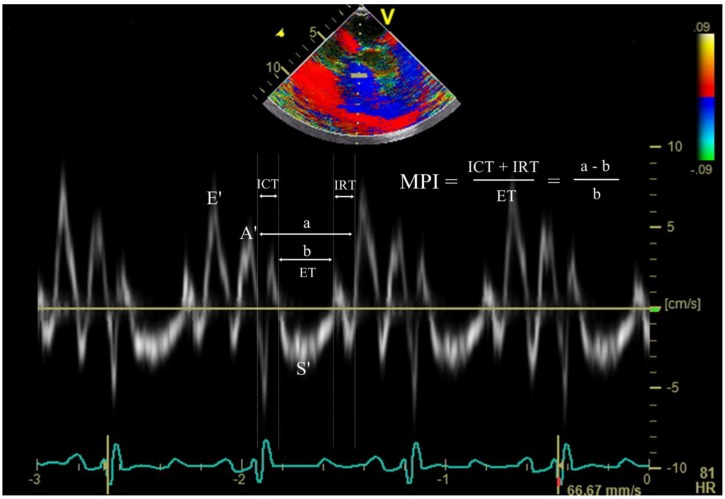
Doppler tissue waves derived from the septal mitral annulus, and time intervals required for calculation of myocardial performance index. E’, peak early diastolic mitral annular velocity; A’, late diastolic mitral annular velocity; S’, peak systolic mitral annulus velocity; ICT, isovolumic contraction time; IRT, isovolumic relaxation time; ET, ejection time; MPI, myocardial performance index.

Hemodynamic variables obtained were the heart rate, mean arterial pressure, and central venous pressure. The echocardiographic assessment was conducted by a single anesthesiologist blinded to the experimental conditions using a single echocardiography system (Vivid E9; GE Vingmed Ultrasound AS) from mid-esophageal four-chamber view at the end of expiration. This assessment included the measurement of LV ejection fraction (LV EF), RV fractional area change (RV FAC), peak early diastolic velocities of septal mitral annulus and lateral tricuspid annulus (MV E′ and TV E′, respectively), late diastolic velocities of MV and TV (MV A′ and TV A′, respectively), peak systolic velocities (MV S′ and TV S′), and tricuspid annular plane systolic excursion (TAPSE). Peak early diastolic transvalvular inflow velocities (E) of MV and TV were also measured to calculate E/E′ for both ventricles. To measure the overall biventricular function, myocardial performance index (MPI) was obtained for the left ventricle and right ventricle from tissue Doppler indices of the mitral valve (MV) and tricuspid valve (TV) using the following formula: MPI = (isovolumic contraction time + isovolumic relaxation time)/ejection time (Figure 1). 

### 2.4. Statistical Analysis

Based on the results from previous studies [8,9] and taking three-way crossover trial into account, sample size was derived from the increase in mean values of P/F ratios from the three PEEP groups (mean values of 170, 210, and 250, respectively, with a standard deviation of 80) [8] while considering mean ± standard deviation values of the peak early diastolic transvalvular inflow velocity (E) of mitral value of conventional Doppler [9]. At a 5% significance level, 36 patients were needed to achieve a power of 80%; hence, six patients for each of the six PEEP combinations were required. A linear mixed model was used for the overall analysis. If statistically significant results were obtained when comparing different PEEP levels, Bonferroni post hoc analysis was conducted. The Doppler data were reanalyzed twice by the first investigator and then once by a second investigator and the intraclass correlation coefficients (ICCs) were calculated with a two-way random model to assess the agreement of the measurements. The strength of agreement was interpreted based on the system proposed by Landis and Koch [10]. Sequence randomization and statistical analyses were performed using Statistical Analysis Software (SAS, version 9.4; SAS, Inc., Cary, NC, USA), and *p* value < 0.05 was considered statistically significant.

## 3. Results

**Figure 2:**
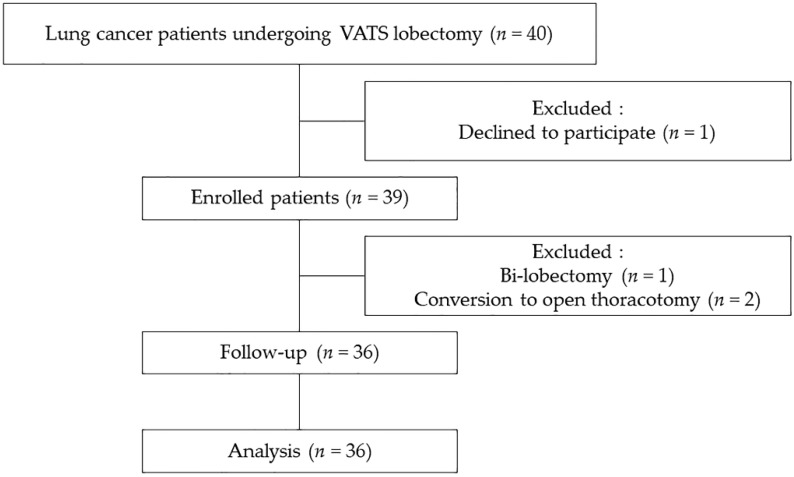
Flow chart of patient enrollment. VATS, video-assisted thoracoscopic surgery.

Of the 40 patients who were screened for the study, four were excluded; therefore, data were collected and analyzed from 36 patients (Figure 2). VATS lobectomy was successfully performed in all 36 patients without any complications during surgery, and all patients were transferred to a general ward through the post-anesthetic care unit. Demographic data are shown in Table 1. 

**Figure 3:**
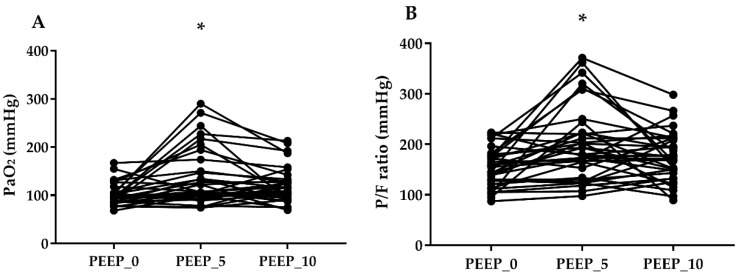
Effects of positive end-expiratory pressure on the changes of arterial oxygen partial pressure (PaO_2_, **A**), the ratio of arterial oxygen partial pressure to inspired oxygen fraction (P/F ratio, **B**). * *p* < 0.05 versus PEEP_0.

PaO_2_ values (*p* = 0.015) and P/F ratios (*p* = 0.014) were significantly different between PEEP_0 and PEEP_5, whereas there was no difference between PEEP_0 and PEEP_10 (PaO_2_, *p* = 0.070; P/F ratio, *p* = 0.051) or between PEEP_5 and PEEP_10 (PaO_2_, *p* > 0.999; P/F ratio, *p* > 0.999) (Figure 3). There were significant differences among the groups in terms of Ppeak and dynamic compliance values (*p* < 0.001), whereas there was no statistical difference in V_D_/V_T_ among the three groups (Table 2).

**Figure 4:**
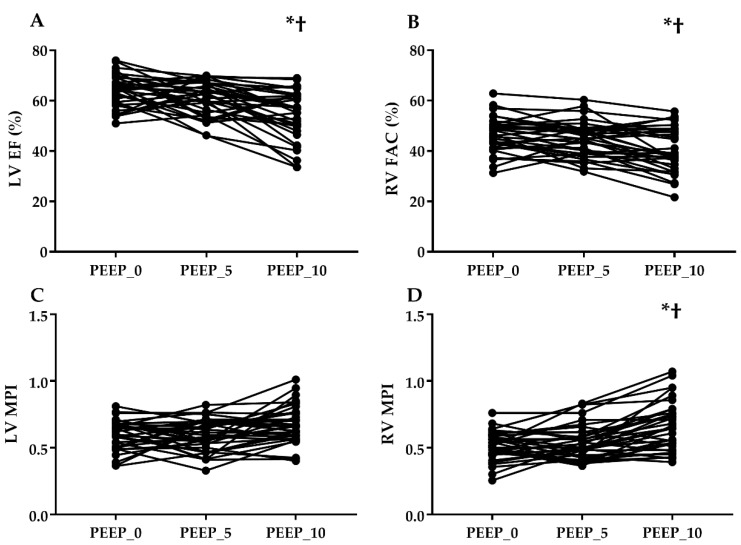
Effects of positive end-expiratory pressure on the changes of cardiac function. (**A**) LV EF, left ventricular ejection fraction; (**B**) RV FAC, right ventricular fractional area change; (**C**) LV MPI, left ventricular myocardial performance index; (**D**) RV MPI, right ventricular myocardial performance index. * *p* < 0.05 versus PEEP_0, † *p* < 0.05 versus PEEP_5.

Hemodynamic variables and tissue Doppler echocardiographic parameters measured at different PEEP levels during OLV are shown in Table 3. Heart rates, central venous pressures, and mean arterial pressures were comparable among the three groups. Both EF and FAC at PEEP_10 showed significant difference compared to PEEP_0 (EF; *p* < 0.001, FAC; *p* = 0.001, respectively) and PEEP_5 (EF; *p* = 0.015, FAC; *p* = 0.023, respectively) (Figure 4). There were no statistical differences among the groups in LV parameters of Doppler echocardiography (MV E′, MV A′, MV S′, LV E/E′). For echocardiographic parameters of the right ventricle, TV E′, TV A′, and TV S′ were comparable among the groups, whereas RV E/E′ values between PEEP_0 and PEEP_10 were significantly different (*p* = 0.044). The results of TAPSE were comparable among the different PEEP levels.

LV MPIs were comparable among three different PEEP levels, while RV MPI value for PEEP_10 (0.64 ± 0.21) was significantly higher than those for PEEP_0 (0.52 ± 0.12; *p* < 0.001) and PEEP_5 (0.54 ± 0.16; *p* < 0.001) (Figure 4).

The ICCs for the agreement of echocardiographic parameters are given in Table 4.

## 4. Discussion

The study evaluated the effects of different PEEP levels on pulmonary oxygenation and biventricular function under OLV. Compared to 5 cmH_2_O of PEEP, cardiac function changed at 10 cmH_2_O of PEEP in both systolic and diastolic phases of left and right ventricles. There was a decreasing trend in biventricular diastolic function as PEEP increased, which was represented by the E/E’ value of both ventricles. The overall RV function decreased when PEEP was increased from 0 to 10 cmH_2_O, which was represented by MPI. However, pulmonary oxygenation was not different between 5 and 10 cmH_2_O levels of PEEP, indicating that higher PEEP level was not advantageous.

General anesthesia with positive pressure ventilation impairs pulmonary gas exchange and respiratory mechanics, even in patients with healthy lungs [11]. Such effects result primarily from the development of atelectasis with subsequent shunting of pulmonary blood flow and impaired oxygenation [12]. During OLV, V/Q mismatch can be aggravated, due to the increase of intrapulmonary shunt [13,14]. Application of positive-end expiratory pressure (PEEP) is an essential part of OLV strategy, since it can overcome V/Q ratio mismatch [13,14] and increase pulmonary compliance [15]. Injury to a patient’s lungs can occur during OLV [16], and the risk for such complication can be reduced by applying low tidal volume with PEEP [4].

There are many conflicting results regarding the effect of PEEP on oxygenation improvement during OLV [17,18,19]. Recently, Ferrando et al. [5] demonstrated that an individualized PEEP, which measured around 10 cmH_2_O, preserved oxygenation and lung mechanics better than consistent 5 cmH_2_O of PEEP during OLV. Several clinical studies on thoracic surgery [20,21] and reviews [1,22] have reported that the adequate level of PEEP should be over 5 cmH_2_O to improve oxygenation during OLV after performing alveolar recruitment. However, other studies showed that 5 cmH_2_O of PEEP with a tidal volume of 6 mL/kg is enough to provide adequate gas exchange [4], improve oxygenation [17], and allow for earlier extubation [23]. A study using a porcine model reported that increasing PEEP to 10 cmH_2_O in healthy lungs did not improve oxygenation compared to that with 5 cmH_2_O [8]. The different effects of PEEP on oxygenation were decided by the difference between total end-expiratory pressure, which was the sum of intrinsic and external PEEP, and the lower inflection point of static compliance curve [15]. PEEP was only beneficial if externally applied PEEP caused total end-expiratory pressure to reach closer to the lower inflection point, otherwise PEEP was associated with a decrease in oxygenation. This study supported that 10 cmH_2_O of PEEP had no beneficial effect compared to 5 cmH_2_O of PEEP in terms of pulmonary oxygenation.

Although the application of PEEP may lead to a reduction in LV afterload, it does not necessarily increase cardiac output, due to the predominant adverse effect on LV filling [24]. Rather, our results showed that LV systolic function decreased when 10 cm H_2_O of PEEP was applied. Increasing levels of PEEP impairs biventricular function [25,26], mainly due to the restriction of the venous return, caused by the elevation of intrathoracic pressure [6]. Furthermore, over 10 cmH_2_O of PEEP was associated with decreased preload, as well as reduced compliance of both ventricles, which was considered to contribute to the changes in diastolic ventricular filling [27]. Even 5 cmH_2_O of PEEP was found to exacerbate LV diastolic relaxation abnormality during TLV in patients with pre-existing diastolic dysfunction [9]. In addition, PEEP influences RV afterload [26], which results in a decreased RV function [28]. Schmitt et al. [26] demonstrated that RV function was significantly impaired, due to the increase in RV outflow impedance when PEEP exceeding 10 cmH_2_O was applied to patients with acute respiratory distress syndrome (ARDS) in the intensive care unit. In OLV, increased right-ventricular afterload augmented by increased airway pressures may provide deleterious increases in shunt fraction and decrease in cardiac output and right-ventricular function [29]. This decrease in RV function may partially account for a decrease in pulmonary flow, leading to a lack of improvement in oxygenation during 10 cmH_2_O PEEP despite improved dynamic compliance. Moreover, the increase in mean alveolar pressure might explain the redistribution of blood flow from the over-distended dependent lung to the non-dependent lung, via increased pulmonary vascular resistance [30].

The indices of tissue Doppler echocardiography are reliable and valuable parameters for estimating intra-operative cardiac functions [31]. It is relatively preload- and afterload-independent [32] and less operator-dependent than two-dimensional or conventional Doppler echocardiography [33]. The MPI, a robust parameter of global ventricular function which is derived from tissue Doppler indices [34,35], has been shown to be a good predictor of perioperative complication in many clinical conditions [36,37]. Indeed, our MPI findings suggest the possibility of RV dysfunction as PEEP increased, which was measured as over 0.55 at PEEP 10 cmH_2_O [38], although these results did not actually lead to changes in hemodynamic variables.

The present study had several limitations. First, we neither returned PEEP to baseline (zero PEEP) nor performed a recruitment procedure during the application of experimental PEEP. Nevertheless, a crossover design involving the cyclical application of different PEEP levels was used to offset the blind spot in this study, and no parameter was influenced by previous PEEP application after statistical verification. Furthermore, considering that hypoxic pulmonary vasoconstriction was settled within 10 m after initiating OLV [39], changes in cardiopulmonary function measured 20 m after initiating OLV can be considered significant enough. Second, although the experimental pressures for the current trial were chosen as they had been commonly used under clinical circumstances, further studies are needed to investigate the impact on individualized PEEP. Third, fixing FiO_2_ could have been more appropriate in comparing the effect of different PEEP on pulmonary oxygenation. Indeed, there is a variation in the P/F ratio with the change of FiO_2_ [40]. However, the P/F ratio has been used not only in experimental studies [4], but also in clinical settings to quantify pulmonary gas exchange, including the use in the definitions of ARDS [41]. Fourth, clamping the pulmonary vessel at the cancer lesion may improve V/Q mismatch by reducing the shunt fraction. Since the study was completed before vascular clamping of the non-dependent lung, we think that the impact of lung cancer on the outcome is limited in this study. Finally, the effects of different PEEP levels during OLV on the protection of the lung should be investigated further.

## 5. Conclusions

In conclusion, the results of this study demonstrated that applying 5 cmH_2_O of PEEP augmented oxygenation during OLV without altering biventricular function. However, increasing PEEP to 10 cmH_2_O decreased cardiac function, especially on RV function, without further enhancing pulmonary oxygenation. Based on these findings, physicians should note that there may not be any advantage to applying 10 cmH_2_O of PEEP over 5 cmH_2_O of PEEP during OLV.

## Figures and Tables

**Table 1:** Patients’ characteristics and results of intra-operative data.

	Patients (*n* = 36)
Age (years)	68.2 ± 7.9
Sex (male/female), n (%)	19 (53)/17 (47)
Body surface area (m^2^)	1.7 ± 0.2
Body mass index (kg/m^2^)	24.7 ± 2.9
Smoking history, (None n (%)/Ex n (%)/Current n (%))	23 (64)/13 (36)/0 (0)
Height (cm)	161.4 ± 8.0
Weight (Kg)	64.5 ± 9.8
Lesion, n (%)	
Right upper/middle/lower lobe	13 (36)/5 (14)/6 (17)
Left upper/lower lobe	6 (17)/6 (17)
Type of lung cancer, n (%)	
Adenocarcinoma/squamous cell carcinoma	26 (72)/10 (28)
TNM stage, n (%)	
T1N0M0/T2N0M0/T1N1M0	19 (53)/10 (28)/2 (6)
T2N1M0/T2N2M0/T3N0M0	3 (8)/1 (3)/1 (3)
Preoperative pulmonary function test	
FVC (L)	3.0 ± 0.6
FVC (%, predicted)	97.3 ± 16.8
FEV1 (L)	2.2 ± 0.4
FEV1 (%, predicted)	102.6 ± 15.0
FEV1/FVC ratio (%)	74.4 ± 6.3
D_LCO_ (mL/mmHg/min)	16.4 ± 3.3
D_LCO_ (%, predicted)	94.8 ± 24.2
Intra-operative data	
Duration of anesthesia (min)	190 ± 46
Duration of surgery (min)	133 ± 40
Duration of OLV (min)	111 ± 37
Intake fluid (mL)	1,061 ± 320
Urine output (mL)	246 ± 200
Estimated blood loss (mL)	79 ± 55

Values are expressed as the numbers of patients with percentage, mean ± SD. FVC, forced vital capacity; FEV1, forced expiratory volume in one second; D_LCO_, diffusion capacity of lung for carbon monoxide; OLV, one-lung ventilation.

**Table 2:** Arterial blood gas analysis and respiratory variables during two-lung ventilation (TLV) and one-lung ventilation (OLV) at different levels of positive end-expiratory pressure (PEEP).

	TLV	OLV			*p*-Value
		PEEP_0	PEEP_5	PEEP_10	
pH	7.36 ± 0.04	7.35 ± 0.04	7.35 ± 0.05	7.35 ± 0.04	0.357
Hb (g/dl)	12.0 ± 1.2	11.7 ± 1.6	11.9 ± 1.7	11.8 ± 1.7	0.926
FiO_2_	0.6	0.7 ± 0.2	0.7 ± 0.2	0.7 ± 0.2	0.989
PaO_2_ (mmHg)	208.2 ± 62.4	95.6 ± 27.3	126.6 ± 71.0 *	119.6 ± 59.2	0.012
PaCO_2_ (mmHg)	45.1 ± 4.8	47.4 ± 5.6	47.3 ± 6.1	47.9 ± 6.1	0.377
EtCO_2_ (mmHg)	40.2 ± 4.2	39.0 ± 2.7	39.7 ± 3.3	40.4 ± 3.7	0.188
P/F ratio (mmHg)	327.6 ± 129.8	145.1 ± 53.4	184.0 ± 77.8 *	176.1 ± 77.0	0.010
Ppeak (cmH_2_O)	12.3 ± 3.0	17.8 ± 3.1	19.9 ± 2.8 *	22.9 ± 2.4 *^,†^	<0.001
Dynamic compliance (mL/cmH_2_O)	32.3 ± 10.4	20.9 ± 5.2	24.6 ± 7.3 *	27.5 ± 7.2 *^,†^	<0.001
V_D_/V_T_ (%)	14.9 ± 7.3	18.0 ± 8.0	18.1 ± 8.4	18.8 ± 9.8	0.902

Values are expressed as mean ± SD. * *p* < 0.05 vs. PEEP_0; † *p* < 0.05 vs. PEEP_5. Hb, hemoglobin; PaO_2_, arterial oxygen partial pressure; PaCO_2_, arterial carbon dioxide partial pressure; EtCO_2_, end-tidal carbon dioxide; P/F ratio, PaO_2_/FiO_2_ ratio; Ppeak, peak airway pressure; V_D_, alveolar dead space; V_T_, alveolar tidal volume.

**Table 3:** Hemodynamic and echocardiographic parameters during two-lung ventilation (TLV) and one-lung ventilation (OLV) at different levels of positive end-expiratory pressure (PEEP).

		TLV	OLV			*p*-Value
			PEEP_0	PEEP_5	PEEP_10	
HR (beats/min)		72 ± 13	75 ± 13	73 ± 11	73 ± 12	0.365
MAP (mmHg)		84 ± 15	84 ± 15	86 ± 12	86 ± 15	0.673
CVP (mmHg)		10 ± 3	12 ± 5	13 ± 4	13 ± 3	0.461
LV	EF (%)	58 ± 9	63 ± 9	61 ± 7	55 ± 10 *^,^^†^	<0.001
	MV E’ (cm/s)	5.3 ± 1.5	5.8 ± 1.6	5.6 ± 1.4	5.5 ± 1.1	0.553
	MV A’ (cm/s)	4.9 ± 1.4	5.9 ± 2.4	5.6 ± 1.7	5.2 ± 1.8	0.067
	MV S’ (cm/s)	4.0 ± 0.9	3.9 ± 1.2	3.9 ± 1.1	3.7 ± 0.9	0.473
	LV E/E’	12.3 ± 3.3	12.3 ± 3.1	12.9 ± 3.6	13.6 ± 3.1	0.051
	LV MPI	0.58 ± 0.14	0.60 ± 0.11	0.60 ± 0.15	0.66 ± 0.15	0.054
RV	FAC (%)	44 ± 7	46 ± 10	45 ± 7	41 ± 9 *^,^^†^	0.001
	TV E’ (cm/s)	4.3 ± 1.4	4.9 ± 1.8	4.4 ± 1.6	4.2 ± 2.0	0.129
	TV A’ (cm/s)	6.3 ± 2.5	6.3 ± 2.3	5.6 ± 2.2	5.4 ± 2.2	0.067
	TV S’ (cm/s)	4.7 ± 1.7	4.9 ± 1.8	4.8 ± 1.6	4.3 ± 1.4	0.054
	RV E/E’	10.1 ± 4.0	9.0 ± 3.3	9.7 ± 3.2	10.2 ± 3.0 *	0.048
	TAPSE (mm)	15.8 ± 2.4	15.9 ± 1.8	15.7 ± 2.2	14.9 ± 1.8	0.087
	RV MPI	0.54 ± 0.13	0.52 ± 0.12	0.54 ± 0.16	0.64 ± 0.21 *^,^^†^	<0.001

Values are expressed as mean ± SD. * *p* < 0.05 vs. PEEP_0; † *p* < 0.05 vs. PEEP_5. HR, heart rate; MAP, mean arterial pressure; CVP, central venous pressure; LV, left ventricle; RV, right ventricle; EF, ejection fraction; MV E’, peak early diastolic mitral annular velocity; MV A’, late diastolic mitral annular velocity; MV S’, peak systolic mitral annulus velocity; MV E, peak early diastolic trans-mitral inflow velocity; FAC, fractional area change; TV E’, peak early diastolic tricuspid annular velocity; TV A’, late diastolic tricuspid annular velocity; TV S’, peak systolic tricuspid annular velocity; TV E, peak early diastolic trans-tricuspid inflow velocity; TAPSE, tricuspid annular plane systolic excursion; MPI, myocardial performance index.

**Table 4:** The agreement of measured echocardiographic parameters interpreted by the intraclass correlation coefficients (ICCs).

	Within 1st Investigator	Between 2nd Investigator	*p*-Value
E’	0.977 (0.970–0.983)	0.956 (0.941–0.966)	<0.001
A’	0.987 (0.984–0.990)	0.970(0.960–0.977)	<0.001
S’	0.982 (0.976–0.986)	0.966 (0.944–0.978)	<0.001
E/E’	0.936 (0.916–0.951)	0.920 (0.895–0.939)	<0.001
MPI	0.944 (0.927–0.957)	0.924 (0.901–0.942)	<0.001

Values are expressed as values (95% CI). E’, peak early diastolic annular velocity; A’, late diastolic annular velocity; S’, peak systolic annulus velocity; E, peak early diastolic trans-valvular inflow velocity; MPI, myocardial performance index
